# Correction: Discovery of a heme-binding domain in a neuronal voltage-gated potassium channel

**DOI:** 10.1016/j.jbc.2022.101754

**Published:** 2022-03-02

**Authors:** Mark J. Burton, Joel Cresser-Brown, Morgan Thomas, Nicola Portolano, Jaswir Basran, Samuel L. Freeman, Hanna Kwon, Andrew R. Bottrill, Manuel J. Llansola-Portoles, Andrew A. Pascal, Rebekah Jukes-Jones, Tatyana Chernova, Ralf Schmid, Noel W. Davies, Nina M. Storey, Pierre Dorlet, Peter C.E. Moody, John S. Mitcheson, Emma L. Raven

In the JBC research article entitled *Discovery of a heme-binding domain in a neuronal voltage-gated potassium channel* Funding and Additional Information should include British Heart Foundation with grant number NH/18/2/33771 and should read as follows:

This work was supported by 10.13039/501100000268Biotechnology and Biological Sciences Research Council Grants BB/K000128/1 and BB/M018598/1 (to E. L. R.); and by the 10.13039/501100000274British Heart Foundation Grant NH/18/2/33771. This work benefited from the Biophysics Platform of Institute for Integrative Biology of the Cell, supported by 10.13039/100015510iBiSA and by the 10.13039/501100011658French Infrastructure for Integrated Structural Biology (FRISBI) ANR-10-INBS-05.

